# Locating hydrogen positions in the autunite mineral metatorbernite [Cu(UO_2_)_2_(PO_4_)_2_·8H_2_O]: a combined approach using neutron powder diffraction and computational modelling

**DOI:** 10.1107/S205225252100837X

**Published:** 2021-10-02

**Authors:** Fiona M. MacIver-Jones, Polly Sutcliffe, Margaret C. Graham, Carole A. Morrison, Caroline A. Kirk

**Affiliations:** a School of Chemistry, University of Edinburgh, Joseph Black Building, David Brewster Road, Edinburgh, EH9 3FJ, United Kingdom; b School of Geoscience, University of Edinburgh, Crew Building, Alexander Crum Brown Road, Edinburgh, EH9 3FJ, United Kingdom; c Department of Earth Sciences, Natural History Museum, Cromwell Road, London, SW7 5BD, United Kingdom

**Keywords:** neutron powder diffraction, computational modelling, hydrogen positions, metatorbernite, inorganic chemistry, AIRSS, mineral stability, autunite

## Abstract

Metatorbernite [Cu(UO_2_)_2_(PO_4_)_2_·8H_2_O] is a promising remediation material for environmental uranium contamination. Previous X-ray diffraction studies have been unable to definitively locate hydrogen positions within metatorbernite, which are key to determining the hydrogen-bond network that helps to stabilize the structure. Here, hydrogen positions have been determined using a combination of neutron powder diffraction and the computational modelling technique *ab initio* random structure searching (AIRSS). Atomic coordinates determined through Rietveld analysis of neutron powder diffraction data are in excellent agreement with the minimum energy configuration predicted by AIRSS; thus, simulations confirm that our proposed model likely represents the global minimum configuration. Two groups of water molecules exist within the metatorbernite structure: free water and copper-coordinating water. Free water molecules are held within the structure by hydrogen bonding only, whilst the coordinating water molecules bond to copper in the equatorial positions to produce a 4 + 2 Jahn–Teller octahedra. The successful agreement between neutron powder diffraction data and AIRSS suggests that this combined approach has excellent potential for the study of other (trans)uranium materials in which hydrogen bonding plays a key role in phase stability.

## Abstract 2

A combination of neutron powder diffraction and *ab initio* random structure searching was used to locate hydrogen positions in the crystal structure of metatorbernite [Cu(UO_2_)_2_(PO_4_)_2_·8H_2_O], a common autunite mineral that shows promise for uranium remediation.

## 1. Introduction   

### 1.1. General   

Autunite minerals are among the most-abundant secondary uranium minerals found in both natural and anthropogenically contaminated environments. Minerals from the autunite group have the general formula *M* ^+^(UO_2_)(*X*O_4_)·*n*H_2_O or *M* ^2+^(UO_2_)_2_(*X*O_4_)_2_·*n*H_2_O (where *M* = Ag^+^, Cs^+^, H_3_O^+^, K^+^, Li^+^, Na^+^, NH_4_^+^, Rb^+^, Tl^+^, or Ba^2+^, Ca^2+^, Cu^2+^, Mg^2+^, Mn^2+^; *X* = P or As; and *n* ranges from 3–12). As autunite minerals incorporate U(VI) within the solid phase, they can minimize the aqueous phase transport of the highly soluble U(VI) species and thus prevent widespread environmental contamination. Consequently, the *in situ* formation of these minerals within permeable reactive barriers has been explored in recent decades as a potential remediation strategy for environmental uranium contamination (Bronstein, 2005 ▸; Lammers *et al.*, 2017 ▸; Szenknect *et al.*, 2020 ▸).

Metatorbernite, a copper-bearing autunite phase with the formula Cu(UO_2_)_2_(PO_4_)_2_·8H_2_O, has been of special interest owing to the common co-occurrence of copper and uranium in primary ores and waste streams. In addition, metatorbernite has a higher thermodynamic stability relative to other autunite minerals (Dzik *et al.*, 2017 ▸, 2018 ▸), uranyl phosphates (Gorman-Lewis *et al.*, 2009 ▸; Shvareva *et al.*, 2012 ▸), uranyl oxyhydrates (Shvareva *et al.*, 2012 ▸), uranyl carbonates (Shvareva *et al.*, 2012 ▸), uranyl silicates (Shvareva *et al.*, 2012 ▸) and uranyl vanadates (Spano *et al.*, 2017 ▸), making it one of the least mobile phases in the environment. Although it is reportedly among the most stable of mineral phases, a better understanding of stability must be acquired before this type of remediation strategy can be implemented on a wide scale, in order to prevent the re-release of uranium into the environment.

Understanding mineral structure is key in predicting stability. The autunite-type structure has been widely studied and is comprised of uranyl-phosphate or uranyl-arsenate sheets stacked along [00*l*] with hydrated interlayers that contain water-coordinated cations, and in some cases, independent groups of square planar water molecules (Beintema, 1938 ▸; Hanic, 1960 ▸; Ross *et al.*, 1964 ▸; Fitch *et al.*, 1983 ▸, 1982 ▸; Locock & Burns, 2003 ▸; Hennig *et al.*, 2003 ▸; Locock *et al.*, 2004 ▸). Of the 50+ structural studies of autunite-group minerals, almost all have been conducted using single-crystal X-ray diffraction (XRD). XRD is a useful technique for locating heavy atoms, such as those in the uranyl phosphate/arsenate sheets; however, the dominant X-ray scattering of uranium means that the majority of studies have been unable to accurately locate the light hydrogen atoms that comprise part of the interlayer water molecules. As hydrogen bonds extend between the autunite sheets and interlayer regions to effectively ‘cement’ the layers together (Fitch *et al.*, 1982 ▸, 1983 ▸; Locock & Burns, 2003 ▸), determining the precise positioning of hydrogen atoms is a key component in predicting the mineral stability.

### 1.2. Previous studies on the metatorbernite structure   

**Figure 1:**
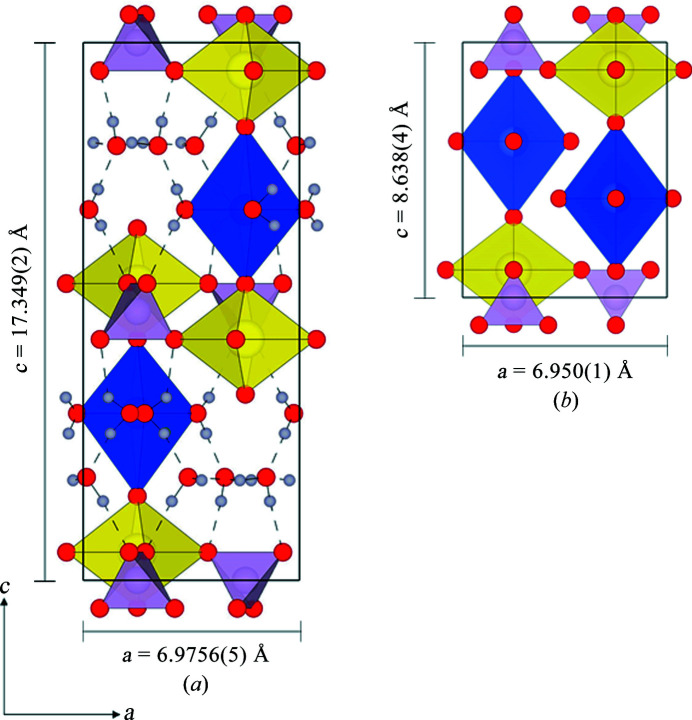
(*a*) An *ac* projection of the structural model of metatorbernite published by Locock & Burns (2003 ▸). (*b*) An *ac* projection of the structural model of metatorbernite published by Calos & Kennard (1996 ▸). Uranyl polyhedra are shown in yellow, phosphate tetrahedra in lilac and copper octahedra in blue; oxygen atoms are displayed as red spheres and hydrogen as light grey spheres. Hydrogen bonds are displayed as dashed lines.

There are several studies that report the structure of metatorbernite. Two different space groups have been suggested, *P*4/*n* and *P*4/*nmm*, with varying *c* parameter dimensions and interlayer structures (Makarov & Tobelko, 1960 ▸; Ross *et al.*, 1964 ▸; Stergiou *et al.*, 1993 ▸; Calos & Kennard, 1996 ▸; Locock & Burns, 2003 ▸; Stubbs *et al.*, 2010 ▸; proposed unit cells are summarized in Table S1 of the supporting information). Fig. 1 ▸(*a*) shows the *ac* projection of the metatorbernite structure as described by Locock & Burns (2003 ▸) in *P*4/*n* and Fig. 1 ▸(*b*) shows it as described by Calos & Kennard (1996 ▸) in *P*4/*nmm*. The general structure is understood to contain uranyl-phosphate autunite-type sheets stacked in the [001] direction. The interlayer regions contain water-coordinated Cu^2+^ cations and independent groups of free water molecules on crystallographically unique sites. The Cu^2+^ cation is octahedrally coordinated, but is Jahn–Teller distorted, with four short equatorial bonds to coordinating water molecules in a square planar arrangement, and two longer axial bonds to uranyl oxygen atoms in both the sheet above and below. The free water molecules also exhibit a square planar arrangement, whereby each oxygen atom is situated at a corner, held within the structure by hydrogen bonds only. Models typically contain one fully occupied Cu^2+^ site, with the free water molecules occurring in either the plane above or below, alternating in the [001] direction as shown in Fig. 1 ▸(*a*). Calos & Kennard (1996 ▸) have alternatively suggested a smaller unit cell, with a *c* parameter half that of the other models and a statistically half-occupied copper site, Fig. 1 ▸(*b*). The phosphate tetrahedra and metal-centred octahedra are rotated relative to the model presented in Fig. 1 ▸(*a*). The authors were unable to establish hydrogen positions.

Despite consensus regarding the general autunite-type structure, atomic coordinates for copper and oxygen atoms vary significantly across the literature. The variation between structural models could, in part, be due to a range in the crystal quality used for analysis. For example, Ross *et al.* (1964 ▸), Stergiou *et al.* (1993 ▸), Calos & Kennard (1996 ▸), and Stubbs *et al.* (2010 ▸) used natural samples obtained from different localities. Stergiou *et al.* (1993 ▸) showed a copper-deficient stoichiometry [Cu_0.9_(UO_2_)_2_(PO_4_)_2_·8H_2_O], while Stubbs *et al.* (2010 ▸) showed a partial replacement of P by As on the tetrahedral sites, an impurity known to alter the unit-cell dimensions of metatorbernite (Kulaszewska *et al.*, 2019 ▸). In contrast, Locock & Burns (2003 ▸) synthesized single crystals for their structural studies. While the synthesis prevented incorporation of arsenic and other impurities, they too described a copper-deficient stoichiometry [Cu_0.88_(UO_2_)_2_(PO_4_)_2_·8H_2_O], with the possible substitution of Cu^2+^ for H_3_O^+^. Furthermore, as all of the aforementioned studies were performed using XRD, the variation between structural models is probably also due to limitations induced by the dominant X-ray scattering of uranium and the associated difficulty in accurately locating lighter atoms (*e.g.* O and H) in the structure.

Only one study has been able to predict potential hydrogen positions within the metatorbernite structure. Locock & Burns (2003 ▸) used difference Fourier maps from analysis of single-crystal XRD data to estimate hydrogen positions and derive a network of hydrogen bonds, as shown in Fig. 1 ▸(*a*). Not only did the suggested hydrogen bonds show how the free water groups may be held within the structure, but also how hydrogen bonds may link between sheet and interlayer to aid in overall crystal stability. However, their suggested H–O–H bond angles of 76° would be highly energetically unfavourable (Milovanović *et al.*, 2020 ▸), and so the requirement for further investigations remains.

Herein we have re-examined the structure of metatorbernite [Cu(UO_2_)_2_(PO_4_)_2_·8H_2_O] using neutron powder diffraction (NPD) to investigate whether analysis of NPD data can provide a structural model with a more feasible configuration of interlayer hydrogen atoms. As neutrons primarily interact with atomic nuclei rather than electron density, NPD analysis does not suffer from the same limitations as XRD with regards to locating light atoms. In addition to these experiments, *ab initio* random structure searching (AIRSS) was performed to evaluate potential low-energy structures (Pickard & Needs, 2011 ▸). AIRSS is a powerful computational modelling tool for structure prediction and involves the generation of numerous models which are then relaxed to nearby local energy minima in order to determine the most likely low-energy structure. Using this combined approach, we aim to provide an accurate model for the interlayer structure of metatorbernite, to ultimately allow for more accurate predictions of the mineral stability.

## 2. Experimental   

### 2.1. Synthesis   

Metatorbernite was synthesized via the aqueous precipitation method as detailed by Cretaz *et al.* (2013 ▸). A uranyl nitrate solution was prepared by dissolution of 3 g of uranyl nitrate hexahydrate in 0.2 *M* nitric acid, followed by the addition of 0.6 g of copper nitrate trihydrate and 3 ml of 2 *M* phospho­ric acid. After 48 h of maturation, the resulting pale green precipitate was separated from the supernatant via vacuum filtration, washed three times with deionized water and dried at room temperature. Once dried to a constant weight, the sample was ground into a fine polycrystalline powder using an agate pestle and mortar. Phase purity and crystallinity were confirmed using powder X-ray diffraction (PXRD), scanning electron microscopy (SEM) with energy-dispersive X-ray analysis (EDS), and inductively coupled plasma optical emission spectroscopy (ICP-OES). The sample was then deuterated via repeated reflux in excess D_2_O (Merck, D 99.9 atom %) at 75°C until complete deuteration was confirmed via Fourier transform infrared (FTIR) spectroscopy. The deuterated sample was dried under a nitro­gen atmosphere to prevent D_2_O–H_2_O exchange, then loaded into a stainless-steel sample cell and sealed for transport and analysis.

### 2.2. Sample characterization   

Phase purity and crystallinity were analysed pre- and post-deuteration via PXRD using a BRUKER D2 PHASER diffractometer (Cu *K*α radiation, λ = 1.5418 Å) equipped with a LYNXEYE detector in Bragg–Brentano geometry. Data were recorded at room temperature over the 2θ range 5° < 2θ < 60° with a step size of 0.03° and a counting time of 14 min. The sample was covered with a Mylar thin film (Chemplex) to prevent spillage of radioactive material.

Elemental composition and sample stoichiometry were determined using SEM-EDS and ICP-OES. SEM-EDS analyses were conducted using a Carl Zeiss SIGMA HD VP FEG-SEM (high-definition variable-pressure field-emission-gun-SEM) instrument with an Oxford Instruments AZtec EDS system at an operating voltage of 15 kV. The Cu:U:P stoichiometry was determined via ICP-OES using a Perkin Elmer Optima 5300DV instrument (selected wavelengths are available in Section S1 of the supporting information). The sample was prepared via digestion in concentrated nitric acid (15 ml; AnalaR 70%). Dissolved samples were boiled down to 1 ml then diluted to 5 ml using 2% HNO_3_ (AnalaR 70%) to provide a consistent matrix between samples and standards.

Sample deuteration was confirmed by FTIR spectroscopy at room temperature using a Perkin Elmer FTIR Two instrument. Data were collected over a wavenumber range of 4000–500 cm^−1^, focusing on analysis of the ν-OH and ν-OD stretching vibrations. As H_2_O is replaced by D_2_O, the ν stretching band shifts from ∼3450 to ∼2550 cm^−1^, and thus the relative intensities at the two stretching frequencies can be used to qualitatively assess sample deuteration (Čejka *et al.*, 1984 ▸).

### 2.3. Neutron powder diffraction   

NPD data were collected at the UK spallation source ISIS, Rutherford Appleton Laboratory, on the POLARIS instrument (Smith *et al.*, 2019 ▸). Approximately 3.5 g of metatorbernite powder was loaded to a depth of ∼20 mm into an 11 mm diameter thin-walled stainless-steel sample cell, which was connected to a gas-handling panel for subsequent data collection during dehydration at elevated temperatures (MacIver-Jones *et al.*, in preparation). A cylindrical collimator manufactured from boron nitride with 8 mm wide slits for the incident and scattered neutron beams was fitted to the outside of the sample cell and enabled data to be collected in the POLARIS 2θ ≃ 90° detector bank (bank 4), which was free of Bragg reflections from the steel of the cell. This assembly was then placed into a furnace mounted on the beamline. Diffraction data were collected at room temperature (∼18°C) for a 250 µA h proton beam to the ISIS target, corresponding to ∼1.5 h total exposure time (Kirk *et al.*, 2019 ▸).

Data collected at room temperature on the 2θ ≃ 90° detector bank was used to carry out Pawley and Rietveld refinements using the *TOPAS Academic* suite of crystallography programmes (Coelho, 2012 ▸). Pawley refinements were used to confirm the space group and unit-cell parameters of the sample, and to ensure that values were consistent with the literature (Makarov & Tobelko, 1960 ▸; Ross *et al.*, 1964 ▸; Stergiou *et al.*, 1993 ▸; Calos & Kennard, 1996 ▸; Locock & Burns, 2003 ▸; Stubbs *et al.*, 2010 ▸). Rietveld refinements were then carried out using the refined unit-cell parameters obtained from Pawley analysis and starting atomic coordinates from the model proposed by Locock & Burns (2003 ▸), Inorganic Crystal Structure Database (ICSD) collection code 97286.

### 2.4. *Ab initio* random structure searching   

The lowest energy positions of the 36 hydrogen atoms in the metatorbernite unit cell were located in a non-biased way through an iterative series of AIRSS/*CASTEP* 19.11 geometry optimization calculations (Clark *et al.*, 2005 ▸; Pickard & Needs, 2011 ▸), with the unit cell and heavy-atom positions taken from the structure reported by Locock & Burns (2003 ▸). Structures were created by positioning two hydrogen atoms randomly on a sphere of radius 1.0–1.2 Å around each oxygen atom designated as a water molecule, with a minimum distance constraint of 1.2 Å between hydrogen atoms applied to minimize the probability of molecular H_2_ forming in the unit cell. The resulting structures were then optimized (atomic positions only) by *CASTEP* (basis-set energy cut-off: 700 eV; DFT functional: Perdew–Burke–Ernzerhof; pseudopotentials: on the fly; minimum *k*-point spacing: 0.05 Å^−1^) until the following criteria were satisfied: energy tolerance, 5 × 10^−4^ eV; maximum force tolerance, 0.075 eV Å^−1^; and atomic displacement tolerance, 5 × 10 × 10^−2^ Å.

## 3. Results   

### 3.1. Phase purity and stoichiometry   

PXRD data collected on the deuterated sample were consistent with data collected pre-deuteration and with the ideal reflection positions for metatorbernite in space group *P4*/*n* obtained from the ICSD (collection code 97286). PXRD data are presented in Fig. S1 of the supporting information. Minor occurrence of the dodecahydrate phase torbernite [Cu(UO_2_)_2_(PO_4_)_2_·12H_2_O; ICSD collection code 97284; Locock & Burns, 2003 ▸] was identified in the PXRD data post-deuteration and was thus included in the refinement strategy. ICP-OES analysis carried out pre- and post-deuteration revealed Cu:P:U ratios of 0.99 (8):2.0 (1):2.06 (2) and 1.00 (3):2.0 (1):2.02 (9), respectively, consistent with the ideal ratio for metatorbernite (1:2:2) and thus indicating that no significant substitution of Cu^2+^ for H_3_O^+^ had occurred during synthesis or reflux. No elemental impurities were detected by SEM-EDS (data provided in Fig. S2).

### 3.2. Sample deuteration   

**Figure 2:**
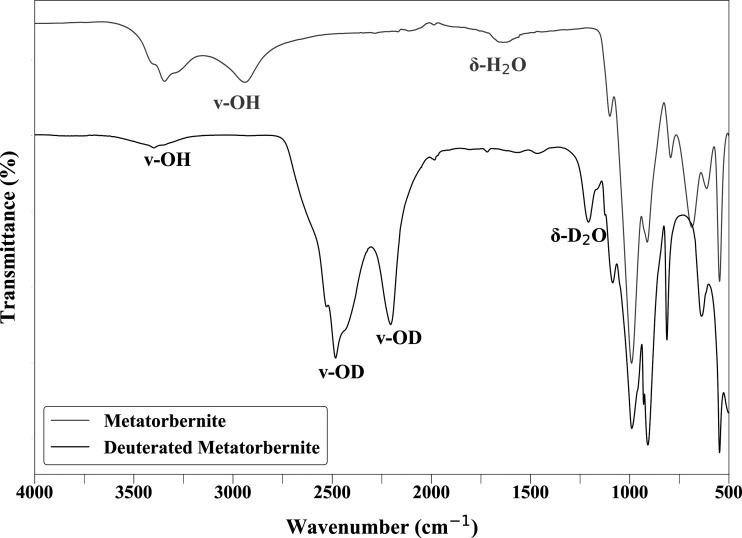
Infrared spectra of metatorbernite pre- and post-deuteration (shown as grey and black, respectively), with labelled ν-DO and ν-HO stretching bands and δ-D_2_O and δ-H_2_O bending bands.

FTIR data collected for metatorbernite pre- and post-deuteration are displayed in Fig. 2 ▸. Two discrete ν-OH stretching bands were observed for the start material, at ∼3350 and 2900 cm^−1^, as also described by Suzuki *et al.* (2005 ▸) for natural metatorbernite. Two discrete ν-OD stretching bands were also observed in the deuterated sample, at ∼2488 and 2220 cm^−1^. The ν-OD stretching bands were observed at lower wavenumbers compared with the ν-OH stretching bands owing to the greater atomic mass of deuterium relative to hydrogen. The two discrete stretching bands can be attributed to two unique water bonding environments in the crystal structure [Suzuki *et al.*, 2005 ▸; Fig. 1 ▸(*a*)]: free non-coordinating water molecules and copper-coordinating water molecules. The free water molecules have strong O–H/O–D bonds as they are not influenced by coordination. We therefore observe the respective ν-OH/ν-OD stretching band at the higher frequency (∼3350 and 2488 cm^−1^, respectively). In contrast, copper-coordinating water molecules are influenced by the Cu–O bonds, which weaken the strength of O–H/O–D and lowers the frequency of the observed ν-OH/ν-OD stretching (∼2900 and 2220 cm^−1^, respectively). The ν-OD stretching bands displayed a greater relative intensity compared with the ν-OH bands in the start material, as similarly observed by Falk & Knop (1977 ▸) in crystalline hydrates (deuterated and non-deuterated). A minor band at ∼3400 cm^−1^ was observed in the FTIR data post-deuteration, corresponding to ν-OH stretching of the free H_2_O molecules (Suzuki *et al.*, 2005 ▸), thus suggesting minor D_2_O–H_2_O exchange had occurred prior to sample loading.

The δ-H_2_O and δ-D_2_O bending vibrations were observed at 1630 and 1190 cm^−1^, respectively. The broadness of the δ-H_2_O band and shouldering of the δ-D_2_O band also indicate the presence of two unique bonding environments. Bands in the lower-wavelength region (∼1170–500 cm^−1^) can be assigned to phosphate and uranyl groups, as fully described by Čejka *et al.* (1984 ▸).

### 3.3. Structural refinement   

Rietveld refinement of NPD data was carried out using the starting model for metatorbernite described by Locock & Burns (2003 ▸) (ICSD collection code 97286) in the space group *P*4/*n*. The model required replacement of H with D due to the significantly different coherent neutron-scattering lengths of the two isotopes (Sears, 1992 ▸). The background was modelled using a Chebyschev function, followed by refinement of the unit-cell parameters, instrument parameter DIFA (Smith, 2011 ▸) and profile parameters using a time-of-flight specific Lorentzian peak shape to model sample contribution. Because the FTIR spectrum indicated the minor presence of H_2_O, H was included in the refinement by locating H and D on certain sites, and their occupancies were refined. Atomic positions and isotropic thermal parameters (Beq) were refined to convergence, then the atomic occupancies of H and D were refined again. Distance restraints with a tolerance setting of 0.01 Å were used for D–O bonds (∼0.99 Å), P–O bonds (∼1.53 Å) and axial U–O bonds (∼1.79 Å) (Burns *et al.*, 1997 ▸; Locock & Burns, 2003 ▸; Locock *et al.*, 2004 ▸; Soper & Benmore, 2008 ▸; Persson *et al.*, 2018 ▸). In several cases, discrepancies were observed between the Beq values for the same element on different crystallographic sites. These Beq parameters were therefore constrained during refinement in order to give more realistic values (Stubbs *et al.*, 2010 ▸), whilst ensuring no change was observed to the profile fit or the structural model. However, in order to develop a model focusing on the individual hydrogen-bonding environments, the Beq values for each H/D site were refined individually. When the Beq values for H/D in free water [H/D(1) and H/D(4)] and coordinating water [H/D(2) and H/D(3)] were constrained, the value refined to the approximate average of the individually refined values. We chose to include the individually refined Beq values rather than the global average in order to try and better understand the individual sites and their interactions within the hydrogen-bond network.

Torbernite was included as an impurity phase in the initial refinements using the starting parameters obtained from Locock & Burns (2003 ▸) (ICSD collection code 97284), consistent with its minor occurrence as indicated by the PXRD data. However, this addition provided only a minor improvement of fit and accounted for just ∼1.6 wt.% of the sample. As torbernite is not typically stable at room temperature, it was assumed that the phase dehydrated to the octahydrate metatorbernite during transport, after the PXRD data was collected. As the proposed contribution was below the typical experimental detection limit of 2–3% (Schofield *et al.*, 2002 ▸), torbernite was not included in the final refinement model in order to avoid over-parameterization.

**Figure 3:**
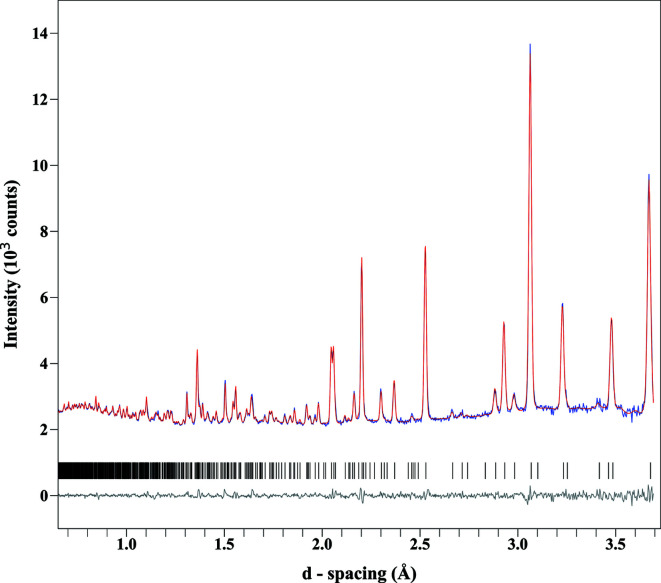
Observed (blue), calculated (red) and difference (grey) profiles after refinement of the NPD data for metatorbernite. Tick marks representing allowed reflections are shown in black.

The final refinement model contained 87 refined parameters with an *R*_wp_ = 2.05 (*R*_wp_ = 1.47 was obtained for the modeless Pawley refinement). The unit cell was refined to give parameters of *a* = 6.9713 (2) Å and *c* = 17.3219 (7) Å. The occupancy of D at sites D(1) and D(4) was found to be 0.80 (1), and at D(2) and D(3) it was found to be 1.00 (3). The refined occupancy of the copper was 1.00 (3), consistent with the stoichiometry obtained through ICP-OES. The observed, calculated and difference profiles are shown in Fig. 3 ▸. Refined atomic coordinates, isotropic displacement parameters and site occupancies are displayed in Table 1 ▸, with bond lengths and angles for water in Table 2 ▸, and selected bond lengths for phospho­rous- and metal-oxygen bonds available in Table S2. Bond valence sums (BVS) were calculated for the final model using the parameters described by Burns *et al.* (1997 ▸) for U(VI) (*R*_0_ = 2.045); by Brown & Altermatt (1985 ▸) for P(V) and Cu^II^ (*R*_0_ = 1.617 and 1.679, respectively); and by Ferraris & Ivaldi (1988 ▸), Grabowski (2000 ▸) and Adams *et al.* (2004 ▸) for H(1)/D(1) (*R*_0_ = 0.925, 0.87 and 2.17 for H⋯O, O–H and O⋯O, respectively). Calculated bond valence values (*vu*) are displayed in Table 3 ▸.

### 3.4. AIRSS   

**Figure 4:**
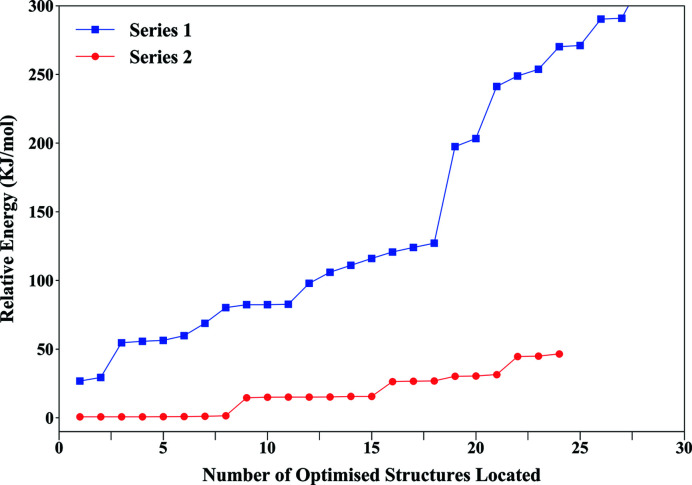
Structure-ranking plots obtained from the two AIRSS runs. Series 1 is shown in blue, where all 36 hydrogen atoms were randomly located on all water molecules; Series 2 is shown in red, where random hydrogen atoms were placed on free water molecules only.

Results obtained from the first AIRSS run, where all 36 hydrogen atoms were positioned randomly, in pairs, around each oxygen atom designated as a water molecule, are shown as an energy-ranking profile in Fig. 4 ▸ (labelled Series 1). This profile shows that the majority of structures obtained lie over a broad energy plateau of *ca*. 50–100 kJ mol^−1^ above the lowest-energy structure, which was found only twice in the data set. Analysis of the lowest-energy structure obtained in this first random search revealed a structure with no overall crystallographic symmetry, indicating a high probability that the lowest-energy structure had not been located. These results indicate that the probability of locating the global minimum structure through the random placement of 36 hydrogen atoms is very low. However, analysing the 11 lowest-energy structures revealed that, while considerable structural diversity existed in the hydrogen-bonding interactions for the eight free water molecules [shown in Fig. 1 ▸(*a*)], the eight water molecules bound to the copper ions shared common positions. This therefore allowed a confident assignment of half of the total number of hydrogen-atom positions as found in the first AIRSS run. Repeating the AIRSS/*CASTEP* procedure, but now randomizing the positions of the 16 hydrogen atoms in the free water molecular layers only, allowed a new lower-energy structure (by *ca*. 30 kJ mol^−1^, labelled Series 2) to be obtained. Critically, this structure was located eight times out of a total of 24 generated structures, giving confidence that this structure does indeed correspond to the energy global minimum. The space group symmetry for this lowest-energy structure conforms to *P*4/*n*, matching the space group proposed by Ross *et al.* (1964 ▸), Stergiou *et al.* (1993 ▸), Locock & Burns (2003 ▸) and Stubbs *et al.* (2010 ▸).

## 4. Description of structure   

### 4.1. Overall structure   

**Figure 5:**
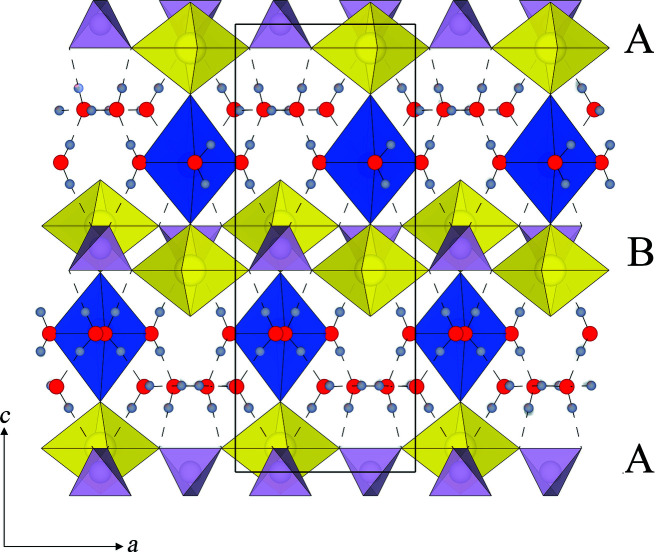
An *ac* projection of the structural model of metatorbernite showing the ABAB stacking arrangement of autunite sheets. Uranyl polyehdra are shown in yellow, phosphate tetrahedra in lilac and copper octahdra in blue; oxygen atoms are displayed as red spheres and hydrogen as light grey spheres. Hydrogen bonds are displayed as dashed lines.

Results from the structural refinement of NPD data and AIRSS are in agreement with published work (Ross *et al.*, 1964 ▸; Stergiou *et al.*, 1993 ▸; Locock & Burns, 2003 ▸; Stubbs *et al.*, 2010 ▸) to suggest that metatorbernite crystallizes in the space group *P*4/*n* with refined unit-cell parameters of *a* = 6.9713 (2) Å and *c* = 17.3219 (7) Å. Our structural model is displayed in Fig. 5 ▸.

The structure of autunite sheets has already been well established using XRD techniques; a full description can be found in Locock & Burns (2003 ▸). Uranyl square bipyramids share equatorial vertices with phosphate tetrahedral units along [100] and [010]. The unit cell contains two symmetrically independent sites for both uranium and phospho­rous atoms, giving rise to two distinct autunite sheets, type A and type B, which are stacked in an ABAB arrangement parallel to [001], as shown in Fig. 5 ▸. The oxygen sites O(1) and O(4), which are axially coordinated to the U(2) and U(1) sites, respectively, extend towards the interlayer regions creating bonds with the Cu^2+^ cations, which form the axial vertices of Cu octahedra (further detailed in Fig. S3). The Cu octahedra, which show Jahn–Teller distortion, are equatorially coordinated by four shorter bonds to water molecules [O(7)].

**Figure 6:**
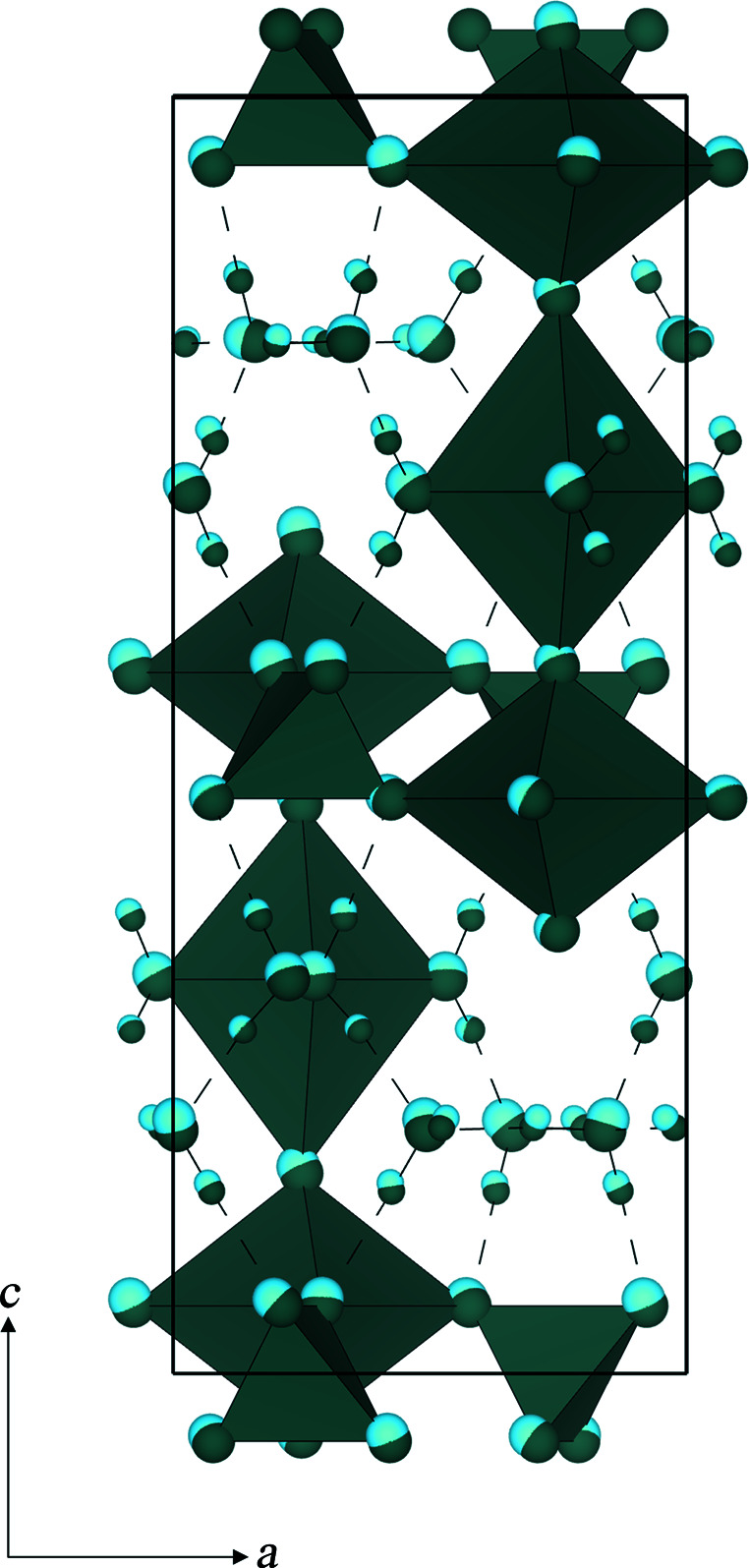
Structure-overlay diagram of the final optimized geometry from AIRSS (shown in light blue) onto the structural model obtained via structural refinement of NPD data (shown in dark teal).

Overlaying the final optimized geometry obtained from AIRSS onto our structural model produced via refinement of NPD data shows a close match was obtained for both heavy atoms and hydrogen-atom positions, as displayed in Fig. 6 ▸. By way of comparison, the root-mean-square deviation of the symmetry-related atoms in the NPD structure and the AIRSS structure was calculated to be just 0.006, indicating a high degree of similarity between atomic positions in the two models. Thus, the simulations confirm that our proposed model is in all likelihood the global minimum configuration for this crystal structure.

### 4.2. Interlayer copper   

The structural model obtained from refinement of NPD data suggests that axial octahedral Cu–O bonds are more asymmetric than previously described, with the refined bond lengths Cu(1)–O(1) at 2.28 (4) Å and Cu(1)–O(4) at 2.73 (4) Å compared with previous models which average around 2.40 Å for Cu(1)–O(1) and 2.65 Å for Cu(1)–O(4) (Ross *et al.*, 1964 ▸; Stergiou *et al.*, 1993 ▸; Calos & Kennard, 1996 ▸; Locock & Burns, 2003 ▸; Stubbs *et al.*, 2010 ▸). This suggests that interlayer Cu^2+^ cations reside closer to one of the autunite sheets than has been proposed in the previous models. Even though Cu is not classified as a light element, its position between the dominant X-ray scattering U atoms has probably led to a less accurate determination of its position in the previous XRD studies. As neutrons interact with the atomic nuclei and are not influenced by the high electron density of the U atoms, we propose that our results reflect a more accurate location of the Cu nuclei. In addition, BVS show that our suggested site satisfies the bond valence for Cu^II^, with a *vu* of 2.08, close to the ideal value of 2.00; whereas in all previous XRD models, the Cu site was either over-bonded with a *vu* ranging from 2.14–2.36 (Locock & Burns, 2003 ▸; Stergiou *et al.*, 1993 ▸; Stubbs *et al.*, 2010 ▸; Calos & Kennard, 1996 ▸; Ross *et al.*, 1964 ▸) or extremely under-bonded with a *vu* of 1.24 (Makarov & Tobelko, 1960 ▸).

### 4.3. Interlayer water   

**Figure 7:**
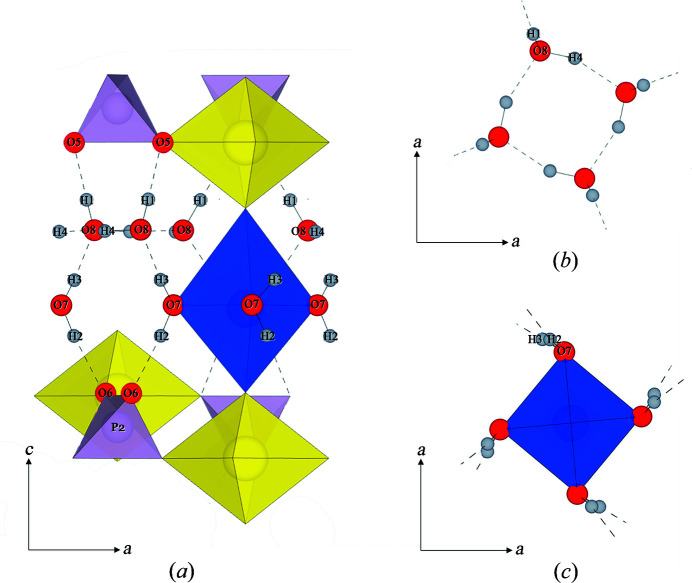
(*a*) An *ac* projection of part of the metatorbernite model, highlighting the configuration of interlayer water molecules and the hydrogen-bond network. Atoms involved in hydrogen bonding are labelled. (*b*) The square planar configuration of free water molecules. (*c*) The square planar configuration of copper-coordinating water molecules.

Fig. 7 ▸ displays our proposed configuration of water molecules obtained from refinement of NPD data. Two independent groups, each containing four water molecules, occupy the interlayer region: free non-coordinating and copper-coordinating water groups. In both groups, the oxygen atoms of the four water molecules are arranged in a square planar configuration, as shown in Figs. 7 ▸(*b*) and 7 ▸(*c*). The free water molecules, H(1)–O(8)–H(4), are non-coordinating and are held within the structure via hydrogen bonds only [Fig. 7 ▸(*b*)]. The copper-coordinating water molecules, H(2)–O(7)–H(3), coordinate Cu^2+^ through O(7) and form the equatorial plane of the octahedral coordination of Cu^2+^ [Fig. 7 ▸(*c*)]. The H–O–H bond angles for the two groups are 109 (2)° and 102 (2)°, respectively, which fit well within the energetically optimal values calculated by Milovanović *et al.* (2020 ▸) for over 40 000 crystal structures (96.41–112.81°) and are close to the ideal bond angles predicted by AIRSS (105.6–105.8°). Our proposed bond angle for the copper-coordinating water molecules is considerably more energetically favourable than previously predicted using X-ray methods at 76 (6)° (Locock & Burns, 2003 ▸).

As deuterium was used as an analogue for hydrogen in our sample, the refined bond lengths in Table 2 ▸ may be marginally lower than those found in hydrogenated metatorbernite. However, as deuteration typically decreases the O–H bond length by only 0.5–3% (Grabowski, 2000 ▸; Soper & Benmore, 2008 ▸), it is believed that any difference in bond length would be minor. It is also worth noting that while two of the four O–H bond lengths fit well within energetically optimal values described by Milovanović *et al.* (2020 ▸) (0.930–0.989 Å), the O(8)–H(4) and O(7)–H(2) bonds sit at the higher end, at 0.99 (2) and 0.99 (3) Å, respectively. These bond lengths are similarly high in – and within error of – those predicted by AIRSS, at 0.999 and 0.987 Å, respectively.

Bond valence values (*vu*) for hydrogen account for the length of the hydrogen bond (H⋯O), as well as the O–H bond length and the distance between the water oxygen and the acceptor oxygen (O⋯O). Calculated *vu* are displayed in Table 3 ▸, using bond distances summarized in Table 2 ▸. All sites reflect near-ideal bonding environments, whereby the *vu* is equal to the valence state of the hydrogen ion, 1. The H(3) and H(4) sites appear slightly over-bonded, with *vu* of 1.10 and 1.09, respectively, while the H(2) site appears slightly under-bonded with a *vu* of 0.93. Nevertheless, the observed over- and under-bonding are minor, and all bond valence values indicate structurally sound hydrogen sites.

### 4.4. Hydrogen-bond network   

Fig. 7 ▸ displays our proposed network of hydrogen bonds. Each free water molecule is involved in four hydrogen bonds: two hydrogen bonds with adjacent water molecules, which link the square planar set together [H(4)⋯O(8)]; one hydrogen bond to a phosphate oxygen in the adjacent autunite sheet, connecting interlayer and sheet [H(1)⋯O(5)]; and one hydrogen bond from the neighbouring copper-coordinating water molecule, connecting the free and copper-coordinating water groups [H(3)⋯O(8)]. The copper-coordinating water molecules participate in only two hydrogen bonds, one to a phosphate oxygen in the adjacent autunite sheet [H(2)⋯O(6)] and one to the oxygen of a neighbouring free water molecule [H(3)⋯O(8)].

Our proposed network of hydrogen bonds is in good agreement with those predicted by Locock & Burns (2003 ▸) using Fourier maps from analysis of single-crystal XRD data. However, several details differ, probably due to differences between the scattering nature of neutrons versus X-rays. Our larger H(2)–O(7)–H(3) bond angle leads to a reduction in the H(2)⋯O(6) bond length by ∼0.16 Å and results in a more favourable hydrogen-bond angle of 174 (1)°, closer to the ideal hydrogen-bond angle of 180° (Jeffrey, 1997 ▸), compared with the previously suggested 138 (6)° (Locock & Burns, 2003 ▸). In fact, all hydrogen bonds in our structural model are shorter, apart from H(3)⋯O(8), which is approximately the same. As both the angle and length of the hydrogen bond influence its strength, these differences have significant implications for mineral stability.

The smallest hydrogen-bond angle in our structural model, *i.e.* furthest from the ideal 180°, occurs between the H(4) and O(8) atoms in the free water groups [157 (1)°], suggesting that these hydrogen bonds are the weakest in the structure (Jeffrey, 1997 ▸). This is also inferred by the high thermal parameter (Beq) of the H(4) site, which indicates vibrational disorder for H(4) atoms. These structural details can be aligned with experimental results to suggest degradation pathways. For example, upon heating of metatorbernite (>70°C), the free water molecules are the first to escape the crystal structure (Suzuki *et al.*, 2005 ▸; Kulaszewska, 2018 ▸), which leads to dehydration and mineral decomposition. The lower strength of the H(4)⋯O(8) bonds suggests that the breakage of these bonds, which stabilize the square planar units formed by H(1)–O(8)–H(4) water molecules, could be the first stage of thermal degradation, followed then by breakage of H(1)⋯O(5) and H(3)⋯O(8), allowing water to leave the structure.

Our results have shown that while X-ray difference Fourier maps can provide a good estimation of hydrogen locations, in order to obtain the detail needed to predict mineral stability, neutron-diffraction studies are required. Verification using computational AIRSS modelling further helps to confirm the structure in terms of energetic favourability. Future work should aim to use this combined approach to investigate other hydrated (trans)uranium phases for which hydrogen bonding is integral to stability. This could include saléeite, a magnesium autunite [Mg(UO_2_)_2_(PO_4_)_2_·10H_2_O] that immobilizes uranium in natural deposits in Koongarra, Australia (Murakami *et al.*, 1997 ▸, 2005 ▸), but for which the proposed hydrogen-bond networks are not in good agreement (Yakubovich *et al.*, 2008 ▸; Dal Bo *et al.*, 2016 ▸); and uranyl peroxide/hydroxide cage clusters, which show exciting promise for the separation and recycling of nuclear fuel, but for which the arrangement of hydrogen atoms and interstitial H_2_O groups has not yet been well established (Burns & Nyman, 2018 ▸).

## 5. Conclusions   

The low electron density of hydrogen has meant that previous XRD studies have been unable to accurately locate hydrogen positions in the autunite crystal structure. Here, we have determined the hydrogen positions in the copper-bearing autunite mineral, metatorbernite, using a combination of NPD and AIRSS computational modelling. Atomic coordinates determined through Rietveld refinement of NPD data are in excellent agreement with the minimum energy configuration as predicted by AIRSS, giving a robust structural model with a detailed network of hydrogen bonds. As hydrogen bonds are a key component in the autunite crystal structure, the accurate determination of hydrogen positions is essential for predicting the mineral stability; with the ultimate aim of determining whether autunite minerals are suitable for the long-term remediation of uranium. Future studies should aim to use this combined approach to investigate further members of the autunite group, as well as other hydrated (trans)uranium phases in which hydrogen bonding plays a key role in phase stability.

## Supplementary Material

Crystal structure: contains datablock(s) metatorbernite. DOI: 10.1107/S205225252100837X/lt5040sup1.cif

AIRSS data. res file. DOI: 10.1107/S205225252100837X/lt5040sup2.txt

Supporting information. DOI: 10.1107/S205225252100837X/lt5040sup3.pdf

CCDC reference: 2102876

## Figures and Tables

**Table 1:** Refined atomic coordinates and isotropic thermal displacement parameters for metatorbernite in *P*4/*n* with unit-cell parameters *a* = 6.9713 (2) Å and *c* = 17.3219 (7) Å

Atomic position	Wyckoff position	Point symmetry	*x*	*y*	*z*	Refined occupancy	Beq (Å^2^)
U(1)	2*c*	4..	0.25	0.25	0.054 (1)		0.5 (1)†
U(2)	2*c*	4..	0.25	0.25	0.550 (1)		0.5 (1)†
P(1)	2*a*	−4..	0.25	0.75	0		0.6 (2)‡
P(2)	2*b*	−4..	0.25	0.75	0.5		0.6 (2)‡
Cu(1)	2*c*	4..	0.25	0.25	0.315 (1)	1.00 (3)	1.2 (5)
O(1)	2*c*	4..	0.25	0.25	0.447 (2)		0.7 (1)§
O(2)	2*c*	4..	0.25	0.25	−0.049 (2)		0.7 (1)§
O(3)	2*c*	4..	0.25	0.25	0.653 (2)		0.7 (1)§
O(4)	2*c*	4..	0.25	0.25	0.157 (1)		0.7 (1)§
O(5)	8*g*	1	0.287 (2)	0.922 (2)	0.0531 (8)		0.9 (1)¶
O(6)	8*g*	1	0.198 (2)	0.925 (2)	0.5494 (8)		0.9 (1)¶
O(7)	8*g*	1	0.531 (2)	0.276 (2)	0.309 (1)		2.2 (3)
O(8)	8*g*	1	0.341 (2)	0.505 (1)	0.8092 (9)		1.4 (3)
D(1)	8*g*	1	0.371 (2)	0.575 (2)	0.8571 (8)	0.80 (1)	0.1 (3)
H(1)	8*g*	1	0.371 (2)	0.575 (2)	0.8571 (8)	0.20 (2)	0.1 (3)
D(2)	8*g*	1	0.581 (1)	0.333 (1)	0.3571 (7)	1.00 (3)	0.9 (3)
D(3)	8*g*	1	0.582 (2)	0.363 (2)	0.2693 (6)	1.00 (3)	2.2 (4)
D(4)	8*g*	1	0.201 (2)	0.479 (2)	0.807 (1)	0.80 (1)	2.6 (5)
H(4)	8*g*	1	0.201 (2)	0.479 (2)	0.807 (1)	0.20 (2)	2.6 (5)

† Beq values for U(1) and U(2) are constrained to be equal.

‡ Beq values for P(1) and P(2) are constrained to be equal.

§ Beq values for uranyl oxygens O(1)–(4) are constrained to be equal

¶ Beq values for phosphate oxygens O(5)–(6) are constrained to be equal

**Table 2:** Interatomic distances (Å) and bond angles (°) for water molecules in metatorbernite

Copper-coordinating water		Free water	
H(2)⋯O(6)	1.92 (2)	H(1)⋯O(5)	1.86 (2)
O(7)–H(2)	0.99 (3)	O(8)–H(1)	0.98 (2)
O(7)⋯O(6)	2.91 (3)	O(5)⋯O(8)	2.85 (2)
O(7)–H(2)⋯O(6)	174 (1)°	O(8)–H(1)⋯O(5)	178 (2)°
			
H(3)⋯O(8)	1.73 (2)	H(4)⋯O(8)	1.73 (2)
O(7)–H(3)	0.98 (3)	O(8)–H(4)	0.99 (2)
O(8)⋯O(7)	2.70 (3)	O(5)⋯O(8)	2.67 (2)
O(7)–H(3)⋯O(8)	172 (2)°	O(8)–H(4)⋯O(8)	157 (1)°
			
H(2)–O(7)–H(3)	102 (2)°	H(1)–O(8)–H(4)	109 (2)°

**Table 3:** Bond valence values (*vu*) for selected atoms in metatorbernite

U(1)	6.04	H(1)	0.98
U(2)	6.02	H(2)	0.93
P(1)	5.02	H(3)	1.10
P(2)	5.01	H(4)	1.09
Cu(1)	2.08		
