# Supplementary material for: Locating hydrogen positions in the autunite mineral metatorbernite [Cu(UO2)2(PO4)2·8H2O]: a combined approach using neutron powder diffraction and computational modelling
Source: IUCrJ. 2021 Oct 2;8(Pt 6):963–72. doi: 10.1107/S205225252100837X (PMC8562655; doi:10.1107/S205225252100837X)
Supplement: Supplementary file 3 [file m-08-00963-sup3.pdf]

IUCrJ

Volume 8 (2021)

Supporting information for article:

Locating hydrogen positions in the autunite mineral metatorbernite [Cu(UO2)2(PO4)2·8H2O]: a combined approach using neutron powder diffraction and computational modelling

Fiona M. MacIver-Jones, Polly Sutcliffe, Margaret Graham, Carole A. Morrison and Caroline Kirk

Table S1 Summary of published unit cell parameters for metatorbernite.

a (Å)	*c* (Å)	Space group	Reference
6.95	17.26	*P 4/nmm*	Makarov & Tobelko (1960)
6.969(1)	17.306(5)	*P 4/n*	Ross *et al.* (1964)
6.972(1)	17.277(8)	*P 4/n*	Stergiou *et al.* (1993)
6.950(1)	8.638(4)	*P 4/nmm*	Carlos & Kennard (1996)
6.9756(5)	17.349(2)	*P 4/n*	Locock & Burns (2003)
6.96519(23)	17.3102(8)	*P 4/n*	Stubbs *et al.* (2010)

S1. Selected wavelengths for ICP-OES

Selected wavelengths for determining Cu:U:P stoichiometry using a Perkin Elmer Optima 5300DV instrument were 224.700 nm for Cu, 177.434 nm for P and 385.958 nm for U. Additional wavelengths of 324.752 nm, 178.221 nm and 409.014 nm for Cu, P and U, respectively, were also analysed and gave near equivalent results to the aforementioned selection.

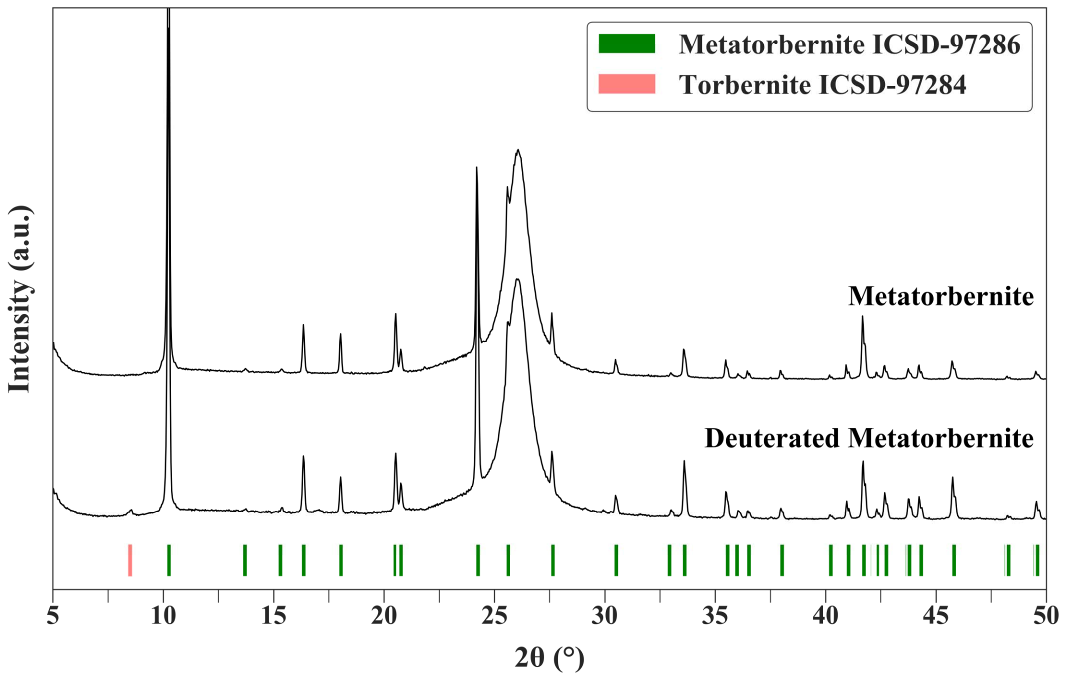

Figure S1 Powder X-ray Diffraction pattern of synthetic metatorbernite pre- and post-deuteration (top and bottom, respectively). Standard patterns shown for metatorbernite in green (ICSD collection code 97286) and Torbernite in pink (ICSD collection code 97286). The Mylar® thin-film is observable via the broad hump centred at 26° 2θ.

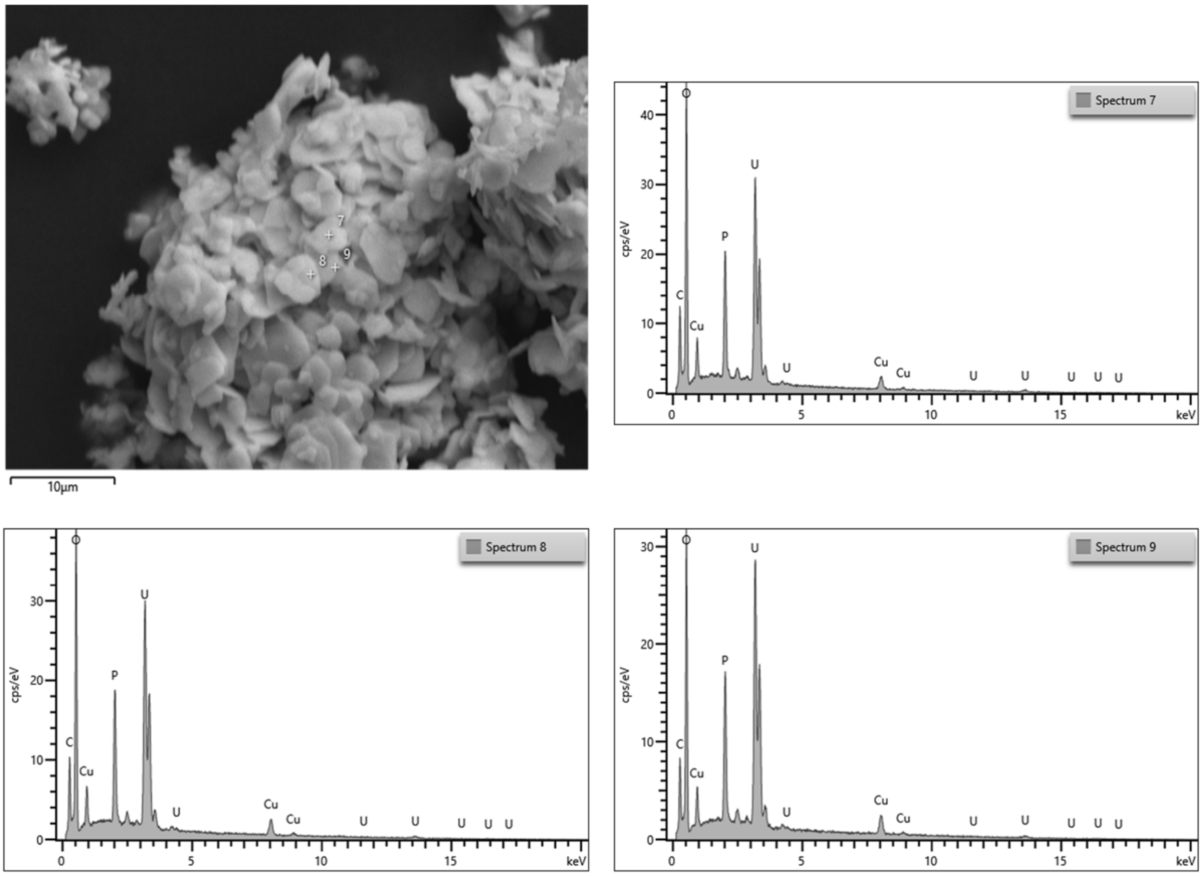

Figure S2 An example SEM image of the metatorbernite start material and several corresponding EDS spectra.

Table S2 Selected phosphorous- and metal-oxygen interatomic distances (Å) for metatorbernite.

U(1)-O(2)	1.79(4)		P(1)-O(5)	1.53(1)	x4
U(1)-O(4)	1.78(3)		P(2)-O(6)	1.53(1)	x4
U(1)-O(5)	2.30(1)	x4			
			Cu(1)-O(1)	2.28(4)	
U(2)-O(1)	1.79(4)		Cu(1)-O(4)	2.73(4)	
U(2)-O(3)	1.79(4)		Cu(1)-O(7)	1.97(2)	x4
U(2)-O(6)	2.30(1)	x4			

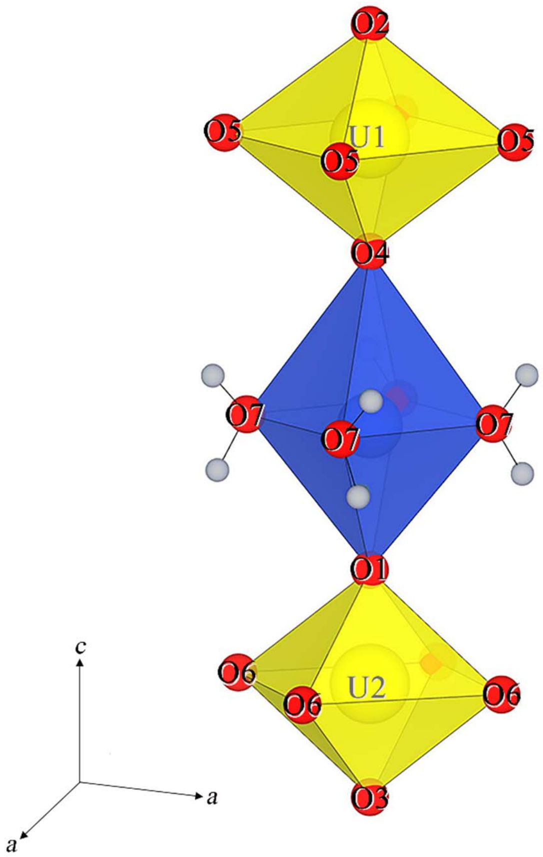

Figure S3 Diagram detailing the bonding between uranyl polyhedra (yellow) and Jahn-Teller distorted copper octahedra (blue) with oxygen atoms shown as labelled red spheres and hydrogen atoms as light grey spheres.
